# Shifting From Sectoral to Integrated Surveillance by Changing Collaborative Practices: Application to West Nile Virus Surveillance in a Small Island State of the Caribbean

**DOI:** 10.3389/fpubh.2021.649190

**Published:** 2021-06-10

**Authors:** Mariana Geffroy, Nonito Pagès, David Chavernac, Alexis Dereeper, Lydéric Aubert, Cecile Herrmann-Storck, Anubis Vega-Rúa, Sylvie Lecollinet, Jennifer Pradel

**Affiliations:** ^1^CIRAD, UMR, ASTRE, Petit-Bourg, France; ^2^ASTRE, CIRAD, INRAE. Univ Montpellier, Montpellier, France; ^3^CIRE Antilles, Santé Publique France, Pointe-à-Pitre, France; ^4^Centre Hospitalier Universitaire, Department of Bacteriology, Virology and Parasitology, Pointe-à-Pitre, France; ^5^Institut Pasteur de Guadeloupe, Laboratory of Vector Control Research, Unit Transmission, Reservoirs and Pathogen Diversity, Les Abymes, France; ^6^Anses, Laboratory for Animal Health, UMR1161 Virology, INRAE, Anses, ENVA, Maisons-Alfort, France

**Keywords:** integrated surveillance, social network analysis, One Health, information system, West Nile virus, Guadeloupe (French West Indies)

## Abstract

After spreading in the Americas, West Nile virus was detected in Guadeloupe (French West Indies) for the first time in 2002. Ever since, several organizations have conducted research, serological surveys, and surveillance activities to detect the virus in horses, birds, mosquitoes, and humans. Organizations often carried them out independently, leading to knowledge gaps within the current virus' situation. Nearly 20 years after the first evidence of West Nile virus in the archipelago, it has not yet been isolated, its impact on human and animal populations is unknown, and its local epidemiological cycle is still poorly understood. Within the framework of a pilot project started in Guadeloupe in 2019, West Nile virus was chosen as a federative model to apply the “One Health” approach for zoonotic epidemiological surveillance and shift from a sectorial to an integrated surveillance system. Human, animal, and environmental health actors involved in both research and surveillance were considered. Semi-directed interviews and a Social Network Analysis were carried out to learn about the surveillance network structure and actors, analyze information flows, and identify communication challenges. An information system was developed to fill major gaps: users' needs and main functionalities were defined through a participatory process where actors also tested and validated the tool. Additionally, all actors shared their data, which were digitized, cataloged, and centralized, to be analyzed later. An R Shiny server was integrated into the information system, allowing an accessible and dynamic display of data showcasing all of the partners' information. Finally, a series of virtual workshops were organized among actors to discuss preliminary results and plan the next steps to improve West Nile Virus and vector-borne or emerging zoonosis surveillance. The actors are willing to build a more resilient and cooperative network in Guadeloupe with improved relevance, efficiency, and effectiveness of their work.

## Introduction

West Nile Virus (WNV) is the world's most widely spread vector-borne flavivirus (1). It primarily affects wild birds, being its natural reservoirs capable of amplifying the virus and maintain it in nature. High-titer viremias develop within infected reservoir birds, which transmit the virus through a cycle involving several mosquito species from the *Culex* genus (2). In addition, WNV can infect several mammalian, avian, and reptile species considered as “dead-end hosts.” WNV (3, 4) is the etiological agent for West Nile Fever in humans, equines, and several bird species, where different disease outcomes of the disease are found, ranging from asymptomatic (in the majority of cases) and mild flu-like illness to severe neurological disease and death (5–7).

WNV was first isolated from the blood of a febrile woman in Uganda in 1937 (3). Initially, the virus was endemic to the African continent and the Middle East with sporadic epidemics in Southern Europe. In the 90s, and even more notably after 2008, more WNV outbreaks were reported in Eastern Europe and the Mediterranean, gaining importance as an emerging and re-emerging pathogen in this region (8).

Several theories suggest that the virus has been mainly spread by migratory wild birds, outside its original distribution range (9, 10). The most striking event regarding WNV diffusion and emergence corresponds to WNV introduction in America from the Middle East in the 90s. A more likely scenario to explain virus introduction in the Americas is through commercial or unintentional transportation of birds or mosquitoes (11).

The New York outbreak marked the first description of WNV on the American continent in 1999 (1), consisting of the onset of severe West Nile Fever cases in horses and humans. Such outbreak was preceded by high numbers of wild bird mortality in the area, putting several public health and veterinary organizations on alert. Subsequently, WNV dispersed northward to Canada and southward to Central America, South America, and the Caribbean (9) following migratory flyways, being the Caribbean situated along the Atlantic and the Mississippi flyways connecting North and South America.

The first detection of WNV within the Caribbean was reported in 2001; a man from the Cayman Islands without a recent travel history (3). In 2002 several islands reported antibody-positive animals, confirming that the virus had arrived in the Dominican Republic, Guadeloupe, Jamaica, and Eastern Mexico (12–14). Viral strains identified in the area were the same as those found in Florida (12). The following year, the virus was circulating in Puerto Rico, Cuba, the Bahamas, and by 2004, the virus was identified in Colombia and on the island of Trinidad (14, 15). More recently, in the British Virgin Islands, a WNV isolate sharing more than 99% nucleotide homology with earlier US WNV strains were found on dead wild birds (Caribbean flamingos, *Phoenicopterus ruber ruber*), confirming that the outbreak resulted from the geographic expansion of US strains (16). Moreover, seropositive equids, both locally bred and imported animals, were found in Saint Kitts and Nevis and Sint Eustatius, meaning WNV was circulating in the Caribbean (17).

In contrast to the situation in North America (7 million WNV human clinical cases recorded since the virus was first detected in the USA), WNV dispersion in Latin America and the Caribbean has been rather silent, without significant bird mortality or clinical manifestations in human and animal populations (11). This has made tracking WNV challenging in the Caribbean region, where the primary information source comes from serological tests. Additionally, there is a high prevalence of other flaviviruses in Central America and the Caribbean; therefore, cross-reactions in serological testing can occur, and positive WNV detections in the area need to be interpreted with care, especially in humans. Lastly, critical wild bird species for virus dispersal and mosquito vector species have not been clearly identified, even though WNV is a public health concern that, due to local meteorological conditions, can be transmitted all year round (10, 18). While the virus has probably become endemic in the Americas, there have been few strains and genomes isolated from outside the USA, with no large WNV outbreaks reported, highlighting the need for further investigation to understand the true burden of WNV in Latin America and the Caribbean (11).

The environmental component is an integral part of the WNV system and is crucial to understand its epidemiological cycle. Several environmental factors - climatic (temperature, rainfall, relative humidity) or other modifications of the habitats or land use can significantly impact vectors and vector-borne disease distribution (19–21). They may indeed play a significant role in the life cycle, distribution, vector and host density (22), and on vector competence (23, 24), influencing the likelihood of vector- virus-host interactions.

Guadeloupe (16°15' N, 61°35' W) is a French Overseas Territory located in the Lesser Antilles in the Eastern Caribbean Sea (22). Differential patterns in precipitation, temperatures, and the fertility of volcanic soil have created highly diverse ecosystems: (i) tropical semi-deciduous forests, (ii) tropical rainforests, and (iii) several types of wetlands like mangroves, lagoons, brackish and freshwater ponds, and swamplands (23, 24). Urbanization and agricultural development also have degraded natural ecosystems. All of these elements can foster WNV vector and host populations. Guadeloupe's main activities are agriculture (banana, sugar cane), and tourism, relying on connections with neighboring Caribbean countries, North America, and France, thus increasing the risks of pathogen introduction, turning Guadeloupe into a hotspot for the emergence and spread of biological hazards.

More than 33 mosquito species are present in Guadeloupe (25, 26), and several vector-borne disease outbreaks (dengue, Zika, and chikungunya) have been recorded in the last two decades. Also, Guadeloupe has about 60 nesting bird species and at least 120 migrant or vagrant bird species (27), making the introduction of WNV through migratory species a real possibility. In July 2002, WNV was identified for the first time in Guadeloupe via serological investigations (ELISA and sero neutralization tests) that revealed the presence of IgG and IgM antibodies against WNV in horses and chicken samples with seroprevalence rates ranging from 2.8 to 10.4%. Six months later, horses' prevalence rate increased up to 50%; which was probably linked to the first WNV incursion in Guadeloupe a couple of months earlier (13). After the first detection of WNV in Guadeloupe, a multidisciplinary surveillance was set up to improve the knowledge of the virus distribution and its burden in human and animal populations of Guadeloupe. As part of the WNV surveillance programs, information campaigns were implemented to increase awareness in physicians, hunters, veterinarians, the general public, and horse and poultry owners. The transmission of WNV decreased dramatically during 2003 and 2004 in horses and poultry. Neither clinical cases in equines nor neurological disorder linked to WNV infections in humans has been reported (18). New seroconversions were detected on horses afterward between September 2007 and August 2008 and between January 2011 and March 2013 (23, 25). Furthermore, mosquito surveillance was set up early in 2015 to monitor mosquito population dynamics in two WNV equine and chicken sentinel sites and to identify the vector species involved (25). Despite these efforts, WNV has never been isolated from mosquitoes, horses, nor bird samples collected in Guadeloupe (28). Additionally, WNV bird mortality was never observed, possibly due to (a) the existence of few susceptible bird species in the territory, (b) hypothetical low vector competence, and (c) possible co-circulation of other flaviviruses (13, 29). In humans, out of nine suspected cases from the Center Hospitalier Universitaire of Pointe-à-Pitre (CHU), none were confirmed as WNV cases, with symptoms that could be attributed to other pathologies. However, it is well-recognized that the causative pathogens of infectious encephalitis in humans in tropical areas are poorly known and investigated (30). Usually, WNV is screened in patients and animals developing neurological symptoms, so mild West Nile Fever (WNF) cases can often go under-recognized (3). Nowadays, WNV surveillance continues in Guadeloupe but mainly involves the veterinary and the entomological components while the actors of the human health sector are in charge of surveillance and control of other human flaviviruses, like dengue and Zika. Most of the WNV surveillance activities in Guadeloupe are sectorial and do not involve regular communication across sectors. After many years of a disassociate surveillance, the actors decided to implement a “One Health” approach to improve WNV surveillance and work more effectively and efficiently together.

WNV surveillance exemplifies how “One Health” approaches can be useful and necessary to understand and create methods for establishing more resilient disease surveillance and control. “One Health” is frequently described as a multidisciplinary and collaborative approach working locally, regionally, and globally to prevent and mitigate risks that originate from the animal-human-environment interface in order to attain optimal health and well-being for everyone (31, 32). There is a particular emphasis on teamwork and communication across disciplines, communities, and sectors, where health problems can be addressed by examining their multiple dimensions. Surveillance using a “One Health” approach, also known as Integrated or “One Health” surveillance, happens when surveillance is organically harmonized, allowing actors and stakeholders from different backgrounds and organizations to work together to control, for instance, a zoonotic pathogen. It consists of systematic collection, validation, analysis, interpretation, and dissemination of information collected on humans, animals, and the environment to inform decisions for more effective, evidence- and system-based health interventions (33). Integrated surveillance aims to share data, providing faster detection and better disease control, compared to sectorial pathogen surveillance, where each sector works only with its data and reacts individually to the outcomes. Benefits have been seen in both disease management efficiency and cost reduction by sharing logistics, human resources, and splitting expenses between institutions. However, joint surveillance is not a problem-free approach due to issues related to barriers for information sharing, unclear responsibilities, privacy regulations, or structural barriers inside organizations adding the lack of communication between actors (33–35). Implementing joint information systems seems necessary and has already been enacted successfully in other parts of the world (36–38).

The application of “One Health” programs aims at offering early detection of circulating WNV in wildlife, mosquitoes, sentinel animals, or confirmed clinical cases. If WNV is detected, human health institutions and authorities are informed and can, in turn, implement vector control measures and preventive activities in the population, thus reducing the number of human infections (38).

Surveillance programs rest on top of two essential pillars: (i) the coordination of the people or organizations in charge of the surveillance and (ii) the establishment of an efficient data exchange network that allows actors from the field to the decision making levels to have quick access to data and information when required (39). The implementation and use of information systems (IS)—defined as “the set of processes implemented to ensure data management” with clear rules concerning sharing mechanisms—is essential for integrated “One Health” surveillance programs.

This article aims to share the experience of Guadeloupean partners who initiated a shift in collaboration practices based on the “One Health” approach applied to the WNV surveillance system in Guadeloupe. This led to the creation of an integrated information system for disease surveillance. Also, a social network analysis (SNA) was implemented in which the relationships, strengths, and weaknesses of the network could be identified, and possible recommendations were made to improve cross-sectoral collaborations and better tackle future emerging zoonotic threats in Guadeloupe in the long-term.

## Materials and Methods

Four participatory workshops were organized between February and November 2020 to which all actors implicated in WNV surveillance were invited. The first two workshops (organized in February and March 2020) had a capacity-building component on Information Systems (IS), databases, and data visualization using R Shiny. The last two workshops (organized in November 2020) focused on testing the new tools, discussing recommendations, and developing a strategic plan toward a more sustainable network.

### Development of the Information System

During the first workshop, the EVASYON method developed by Chavernac, was used following several steps: (i) identification, mapping and role (data collection, centralization, analysis, information sharing) of all the actors and description of surveillance data flow at the regional (Guadeloupe), national (French), and international levels; (ii) identification of current IT resources and data, and creation of a data catalog; and (iii) a comprehensive evaluation of the state of available information and future planning to create an IS for WNV (WNIS) in Guadeloupe. At the end of the workshop, participants agreed on preliminary technical specifications for the creation of an IS (sections, functionalities, users' rights) that would allow participants to collect, store and share data among all participants and organizations that have a role in WNV surveillance, depending on the participants' needs and interests.

A detailed document with all the technical requirements and specifications was prepared by a small working group using an online editable platform to facilitate collaboration with the developer (Google Docs). The WNIS prototype was developed using php/MySQL and the Rapid Application Development of php Runner (https://xlinesoft.com/phprunner). It was adapted to main users' comments and needs shared throughout 2020. A final prototype version was presented to the broader group of WN surveillance actors (veterinarians, DAAF, Santé Publique France, CHU, IPG…) for discussion through another participatory workshop held in November 2020. Improvements have been made to prepare a beta version that will run in 2021 for testing and validation. The final IS will be developed and transferred to a local server using the feedback on its use after several months of field WNV surveillance.

### Data Catalog

Information on the WNV surveillance activities conducted since 2002 by the various institutions was collected, emphasizing that data, reports, and knowledge would be used to develop and test the new WNIS.

The data catalog was prepared using the Dublin Core (40), a metadata structure used to classify electronic resources with a brief description of their content and characteristics. In this case, using the Dublin Core allowed easy creation of the catalog and, in the future, facilitates the proper maintenance, management, and use of existing resources.

### Use of R Shiny for Data Visualization

During the second workshop, partners interested in tool development and conception were trained on R Shiny, an application for dynamic and interactive data visualization entered in the web interface to capture and manage surveillance data reports from the WNIS. Indeed, an attractive and dynamic display of information to the partners and the general audience is one of the goals of this integrated WNV surveillance system. Concurrently to the WNIS development, a graphical and cartographic data visualization web application called VirusTracking was deployed. The application was written in “R” using the Shiny framework. It explores data and information stored in the MySQL database of the IS accessible for the WNV surveillance members from a secured server, and the surveillance databases. It relies on Shiny's reactive programming framework, allowing the communication of results easily via interactive charts, texts, or tables, and compartmentalizes and caches expensive computational stages so that an interactive session does not require calculations and queries to be recomputed unnecessarily. The application is directly connected to the MySQL database and allows the extraction of the information through SQL queries.

### Social Network Analysis

Social Network Analysis (SNA) is defined as a “distinctive set of methods used for mapping, measuring, and analyzing the social relationships between people, groups, and organizations” (41, 42). It can be used to evaluate any network, from businesses, governmental institutions to health and ecological systems that involve people and organizations. In general, knowledge of how actors and partners interact with one another helps understand how the information flows between organizations and under what conditions (43).

Given the large number of organizations belonging to different sectors and backgrounds involved in the WNV surveillance in Guadeloupe, an SNA was used to understand the ties and relationships of the actors implicated in WNV surveillance in Guadeloupe and in identifying the levers and barriers that influence teamwork. Relevant recommendations about desirable changes to increase communication and collaboration will be facilitated.

In order to attain the stated objective, a questionnaire was created and piloted to collect information about the connectivity, centrality, and flux of information between actors. The questionnaire consisted of four parts: (a) personal information, (b) network connections and information flow, (c) actions for the future of the network, and (d) current perception of the network. It also allowed the acquisition of information using focused ethnography, a methodology that helps describe a group, its experiences, attitudes, and interactions (44, 45). Actors were asked to describe and give their opinion about the network's current state, challenges, and future actions for improvement.

Face-to-face, telephone, or via ZOOM™, semi-directed interviews were conducted with the actors involved in the surveillance of WNV. Interviews were done both in French or English, depending on the interviewee's confidence and language management level. All the interviews were recorded for later transcription. If interviews were in French, they were transcribed and translated into English for subsequent analysis.

The qualitative analysis of the actors' interviews was carried out using Nvivo, a qualitative data analysis software (QSR International, Release 1.0). For the network mapping, matrices with actor relationships with one another and their communication level were created. The matrices' analysis was done using R Studio with the following packages: igraph, network, sna, ggraph, visNetwork, threejs, network D3, and ndtv. Map designs were improved using Gephi, an open-source network analysis and visualization software (46).

## Results

The interactions among partners evidenced three critical points: (i) a multitude of persons were involved, (ii) a large set of data was disseminated within the different institutions, and (iii) only a few interactions existed between different actors. Additionally, no single formal WNV surveillance exists, but rather, several surveillance activities are carried out by different institutes that communicate poorly with each other.

### West Nile Surveillance Organization and Available Information/Data

**Figure 1:**
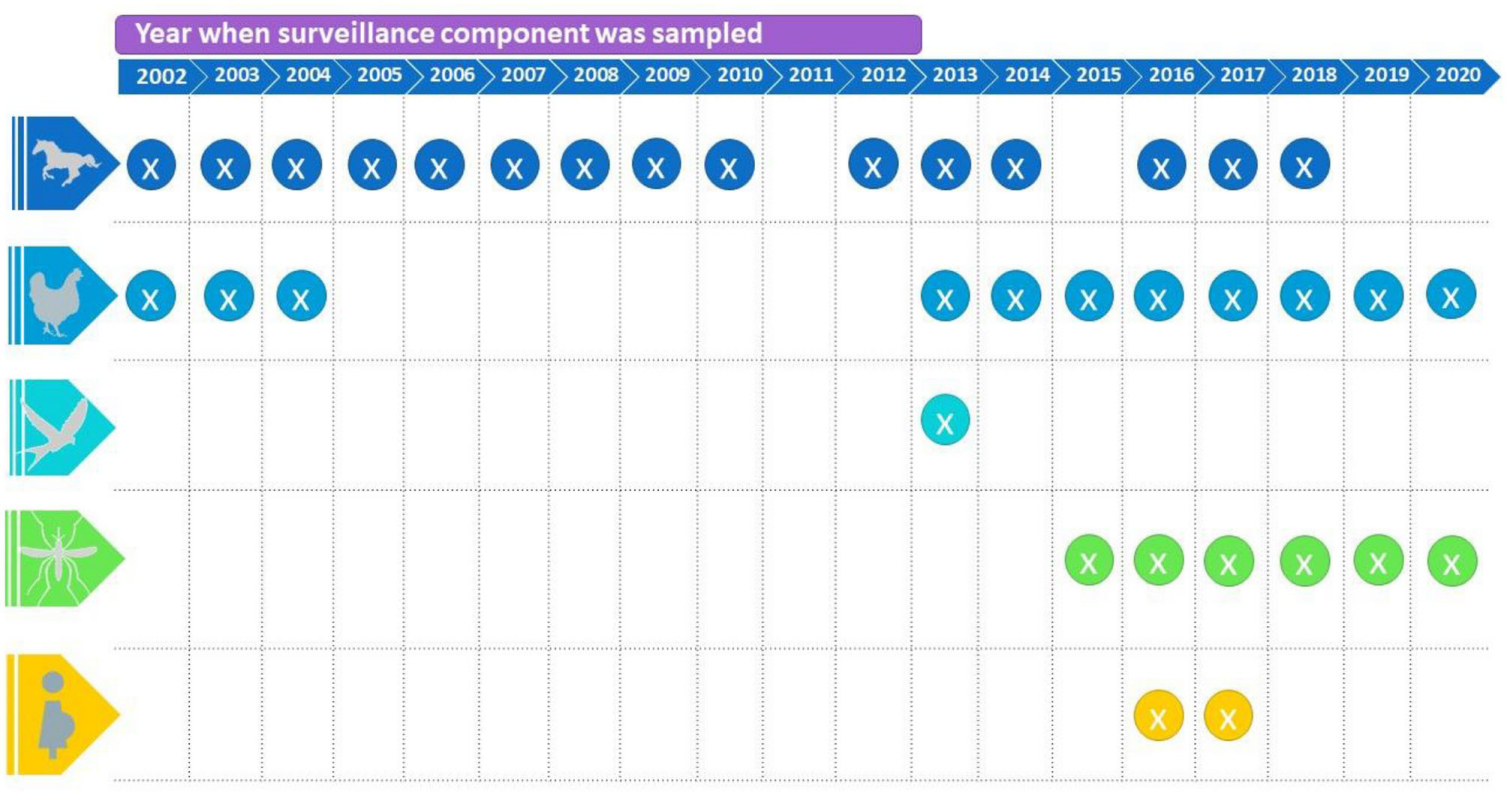
Chronogram of surveillance activities that took place from 2002 to 2020 in horses, poultry, wild birds, mosquitoes, and humans, based on the datasets provided by the partners.

The WNV surveillance organization in Guadeloupe and the data's location is summarized in Table 1 and Figure 1. Every database included information relevant to individual surveillance objectives, and they were centralized at the French Agricultural Research Center for International Development (CIRAD) for future data analysis.

**Table 1:** WNV surveillance activities in Guadeloupe.

**Component of the WNV surveillance**	**Organizations involved**	**Type of surveillance**	**Description of surveillance activities**
Human	CHU Pointe-a-Pitre Interregional Epidemiology Unit of Antilles-Guyane (CIRE) of Santé Publique France (SPF)	Passive	WNV screening in suspected clinical cases: undiagnosed viral encephalitis or meningitis or infections consistent with West Nile Neuroinvasive Disease (WNND). Frequency of data collection: infrequent, highly heterogeneous.
	Regional Health Agency (ARS)	Serosurvey	Flavivirus screening in pregnant women within the framework of Zika surveillance (2016–2017). West Nile was included in the testing.
	National Center of Arboviruses (France)		
Domestic animals (equines and poultry)	Direction de l'Alimentation, l'Agriculture et la Forêt (DAAF) CIRAD Private veterinarians	Active	Use of sentinel equids and chickens to detect WNV circulation. Horses from four sentinel sites were sampled yearly until 2018. Chickens from two sentinel farms were sampled every 15 days between 2013 and 2018 and every 3 months since 2019. Frequency of data collection: regular in chickens some years are missing in horses.
	DAAF CIRAD Private veterinarians	Event-based	WNV screening in suspected clinical cases in equids presenting signs of neurological disease. Frequency of data collection: every year, during the 2nd semester; however, it is not activated correctly every year.
Wild birds	SAGIR network of the OFB CIRAD laboratory	Event-based	Identification of high wild-bird mortality events and testing. Effective in Mainland France. Not operational yet in Guadeloupe.
Wild birds	IPG CIRAD laboratory	Serosurvey	Data collected within the framework of a 2-year project.
Entomological	CIRAD	Active	Mosquito species identification and determination of population dynamics in Guadeloupe. Frequency of data collection: Every 2 weeks since the end of 2014, alternating with the sentinel chicken surveillance in order to be able to detect pools of mosquitoes infected in case of seroconversion observed in poultry.

The primary sources of data came from the following organizations:

CIRAD: Poultry, equine, and mosquito surveillance data. Excel files and Access databases are stored in CIRAD's Laboratory Quality Assurance system and server.

Institute Pasteur of Guadeloupe (IPG): The database of a research project on wild birds was shared. It was organized as a list using the bird capture dates, location, and serological results.

CHU Pointe-à-Pitre: No existing records could be found, but communications were established concerning WNV samplings at the hospital, with concerns about those samples' poor quality, resulting in unreliable results.

Santé Publique France (SPF): A serosurvey of pregnant women was organized in Guadeloupe within the Zika surveillance framework. Several flaviviruses, including WNV, were tested.

The main sources of data were initially organized in separate Microsoft Excel documents. Excel files were further merged by component, completed, harmonized, and validated using the lab result sheets that were delivered to each sampling group. These MS Excel documents were ordered by date, and four MS Excel documents were finalized for each component—equine, birds, humans, and mosquitoes—to prepare future integrated data analysis.

### Pilot WNV Information System and R Shiny component

**Figure 2:**
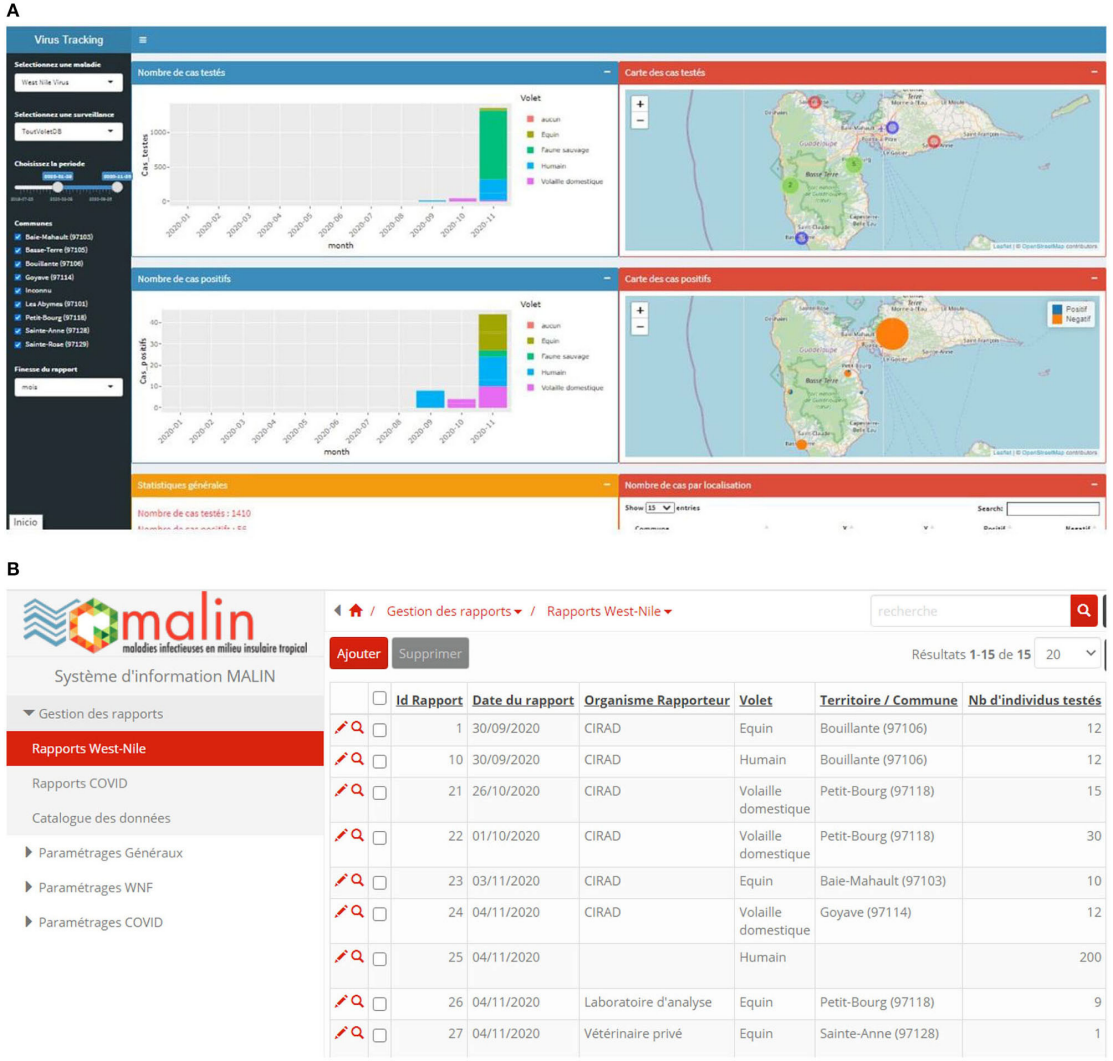
**(A)** Screenshot of the VirusTracking West Nile Virus surveillance platform. The graphs show the reports submitted on the information system on eight communes of Guadeloupe (Baie-Mahault, Basse-Terre, Goyave, Les Abymes, Petit-Bourg, Sainte-Anne, Sainte-Rose) during 2020. The time unit is a month. The data are not real epidemiological data; they were the ones entered by the participants in the workshop for testing the tool. **(B)** Screenshot of the WNIS. WNV report section: The report ID with the organization that submitted the report is listed as well as the component under surveillance, commune, and number of tested and positive cases. The information and data do not correspond to actual data (participants entered them to test the platform).

A consensus was reached among the partners to develop a simple WNIS based mainly on the regular sharing of surveillance reports for the different surveillance components (human, equine, domestic poultry). We hypothesized that each organization had databases already in place and that the IS would not attempt to duplicate data entries in a third-party application but rather allow collecting synthesized data. The main actors agreed that the primary information that would be entered and displayed in the WNIS would be: location (commune), component (human, equine, poultry, wild birds), reporting organization, event, number of individuals tested, number of positive (and test used), number of negative, number of deaths (Figure 2B). The information would be submitted through monthly to quarterly reports. The information is comparable for all surveillance components allowing easy visualization with automatic data recovery routines with R Shiny. The final prototype is being developed using the latest workshops' outputs with the actors who required additional functionalities such as implementing alert flows to notify partners of WNIS activities alongside access rights management. A local web server hosting the platforms and allowing a dynamic connection between the IS and the R-Shiny visualization interface has yet to be identified before the system is passed in production. In the meantime, it is located in a temporary server in mainland France. The demo version is accessible here: https://astre-apps.cirad.fr/apps/tracking-virus/. It showcases information from different surveillance components, displaying both surveillance data (“past” published datasets) and mock surveillance summary reports that were submitted through the WNIS (“current” datasets) with simulated WNV surveillance reports submitted in 2020–2021. The actors are currently testing several options and the system will evolve as new needs arise.

The VirusTracking application is divided into two main panels: the first panel is dedicated to option control (inputs) on the left, and the second panel on the right has graphical data reports (outputs) based on user preferences. Users may first select a disease (WNV) and the species under surveillance (human, equine, avian, wild birds, or mosquito surveillance or all the surveillance components at the same time). Users can then adjust the period and geographical location (i.e., by selecting/deselecting communes) of interest on which they want data to be presented. Data information is then shown and plotted dynamically on the right panel according to the input data user's selection. The resulting panel is organized as follows (Figure 2A):

- Overall statistics of reported tested and positive cases observed in the selected locations during the period.
- Histogram of the number of tests conducted chronologically,
- Histogram of positive cases reported chronologically,
- Guadeloupe region-centric geographical map showing the locations where individuals were sampled.
- Guadeloupe region-centric map reporting positive case vs. individuals tested ratio for each commune. These are represented as mini pie charts whose sizes are proportional to the number of tested cases.

Regarding chronology reports through histogram representation, bar width can be adjusted according to the accuracy required by the analysis, either daily, weekly, monthly, or even by year, to sum up, the information. In practice, this flexibility has been implemented because of the heterogeneous frequency of report acquisition.

The users can also gather information from different surveillance components at once, as different layers overlaid in the same histograms and geographical maps were added. This functionality may be of great importance for helping actors have an overview of the information. This functionality does not substitute Geographic Information Systems that would be helpful to understand WNV epidemiology in Guadeloupe.

### Social Network Analysis

In total, sixteen (16) people out of 20 currently involved in WNV surveillance were interviewed. They were actors participating in all three components of the WNV surveillance (human, animal, and environmental) and belonged to the following institutions: CIRAD, IPG, SPF, CHU Pointe-à-Pitre, the French Agency for Biodiversity (OFB), the Regional Health Agency (ARS), the French Agency for Food, Environmental and Occupational Health & Safety (ANSES), Direction de l'Alimentation, l'Agriculture et la Forêt Guadeloupe (DAAF Guadeloupe), and private veterinarians.

**Figure 3:**
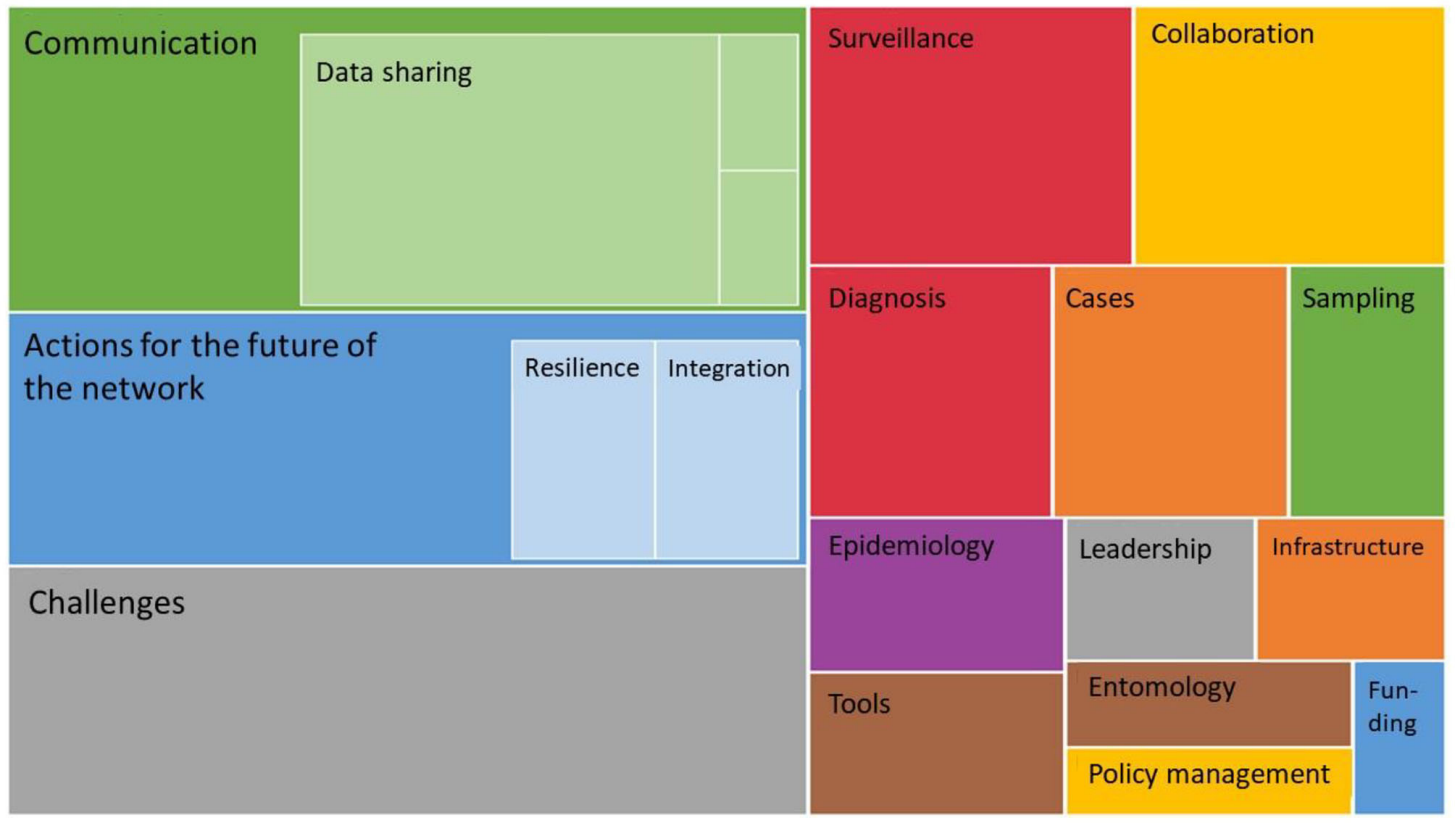
Hierarchy chart of themes obtained from the SNA questionnaire using Nvivo software. The bigger the box, the more frequent the theme.

Actors talked about various themes, but their three main concerns addressed “communication,” “current challenges,” and the “actions for the future” of the network, as can be seen from Figure 3.

**Figure 4:**
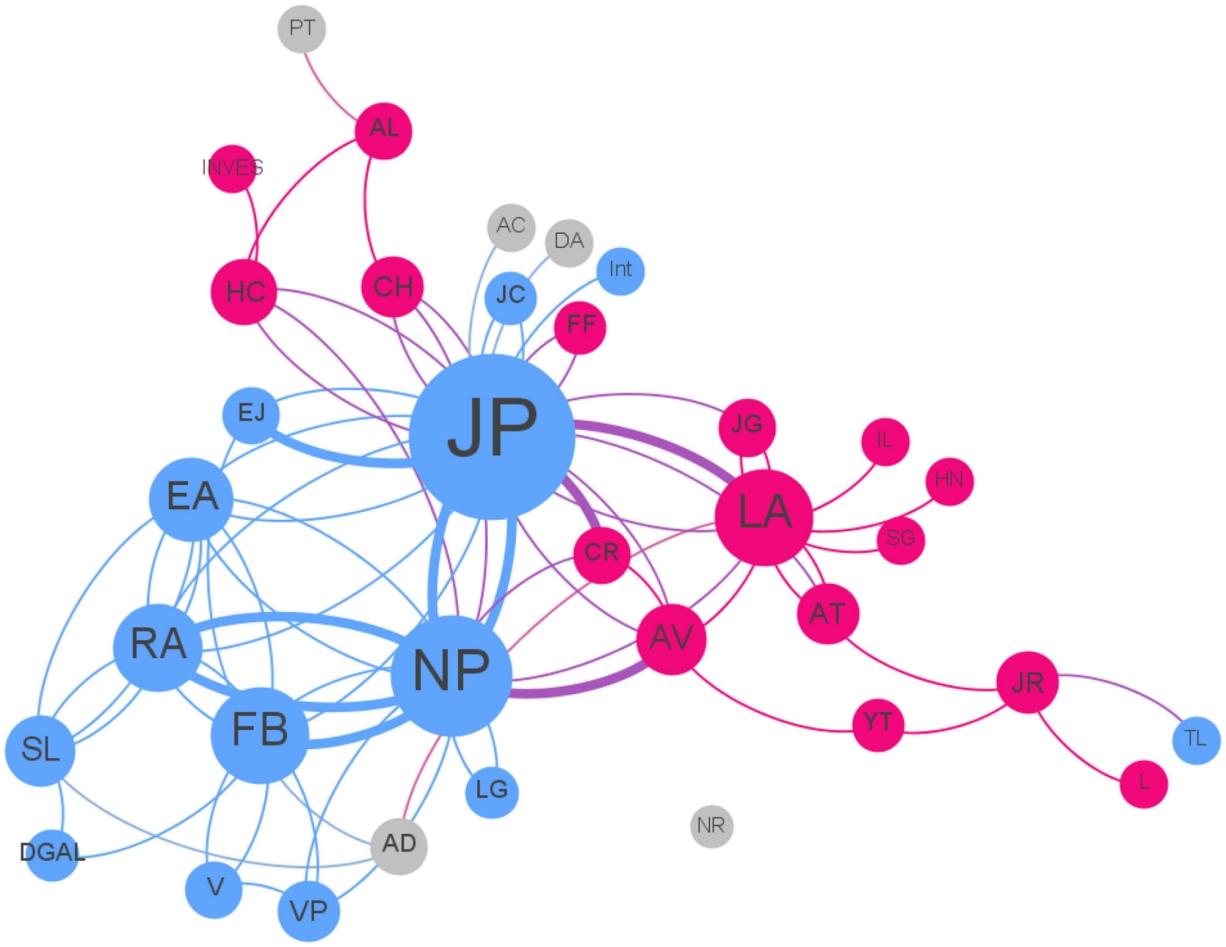
Map of the West Nile Virus surveillance network in Guadeloupe. The colors represent the sectors the actors belong to: animal (Blue), human (Magenta), and the environment (Gray). Larger nodes have more edges. The thicker the lines, the more weight in the communication between two actors.

The network map of the surveillance system (Figure 4) is made up of 36 vertices and 88 edges, having a diameter of six (6), which is the minimum path length that can connect any pair of nodes in the network. The network reciprocity is 52%, meaning that among our pairs of nodes, half of them have a one-way exchange of information. Nearly three-quarters of the nodes (28, 77%) have less than five edges that connect them to other actors of the network. On the other hand, two nodes concentrate 22 and 31 edges (labeled NP and JP, respectively), meaning that more than half (53, 60.2%) of the connections are gathered around these two actors: the main hubs and authorities of information.

**Figure 5:**
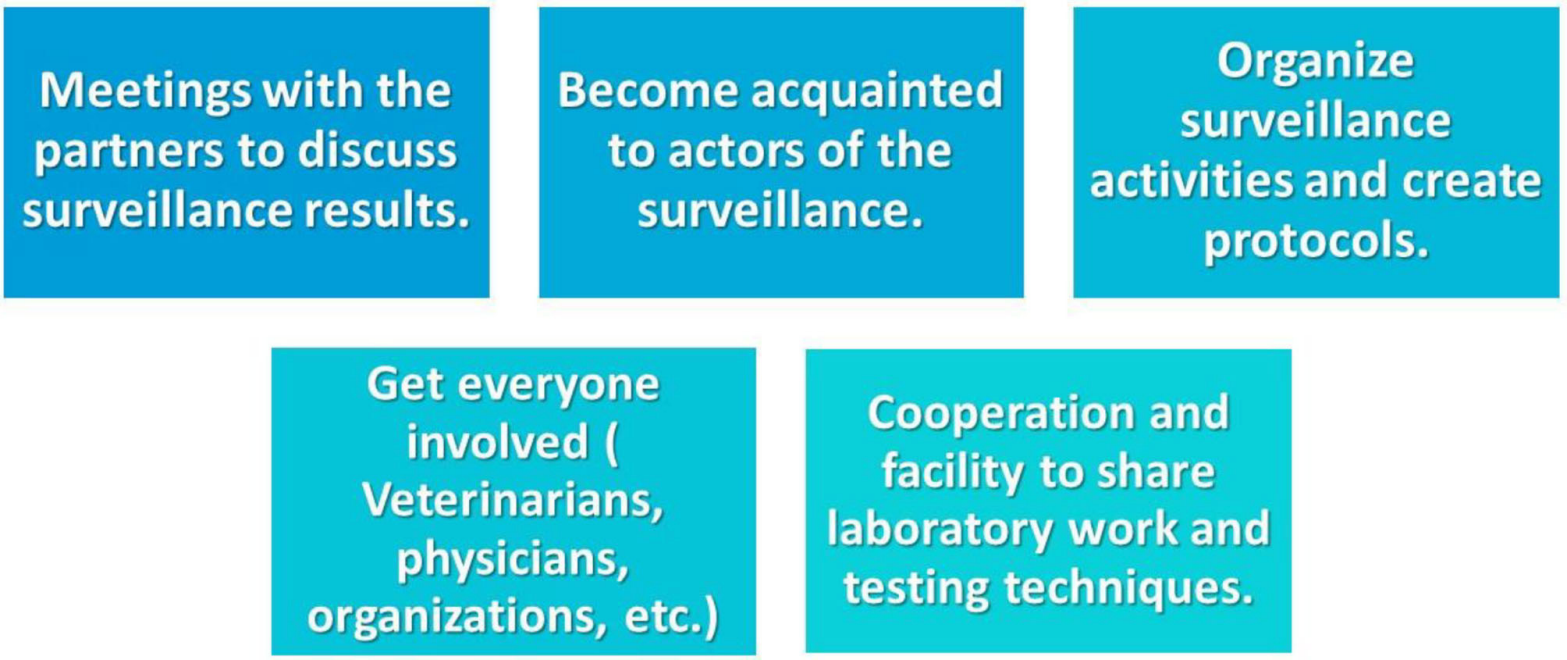
Main actions that were recommended by actors to increase the resilience of the WNV surveillance system.

In addition to data information flows, actors and stakeholders also have provided suggestions and opinions about the levers and barriers of communication inside the system (Table 2), the main challenges (Table 3), and the priorities for improving the surveillance system (Figure 5).

**Table 2:** Constraints and levers for sharing data between actors.

**Information sharing: levers and constraints**	**Actions recommended**	**Examples**
What makes sharing WNV information easy?	• Partners know one another • Trust between partners • Relationships • Existence of available platforms for sharing information (meetings, seminars, newsletters, etc.) • Curiosity • Surveillance is divided, thus needs information from another actor to complete their own.	“*And another point, so I said yes, we know each other, we know what the others are doing, and we may have a common tool, um, possibly the Internet, finally the web, to share minimal information and alerts.”*
What makes sharing WNV information difficult?	• WNV is not a health priority (lack of interest) in Guadeloupe • Information lacks detail • Lack of communication between organizations. • No tools to share information • Lack of cases (human health)	“*Because mainly I think it is not the priority of all people and organizations: they have their own priorities. They are not available for things like this.”*

**Table 3:** Main challenges of the WNV surveillance system with examples.

**Main challenges of the WNV surveillance network**	**Quotes**
No formal network and no existing protocols	“*There is no network. Individual activities are conducted independently from one another. There is no coordination. No established mechanisms for sharing information between the different actors….”*
Undetected transmission. Silent viral circulation	“*I think that with a disease, a flavivirus, you can have a lot of undetected transmissions. WN fever is not a disease that social security tells you to take a test for. That is why people have symptoms, but they are not systematically tested for WN.”*
Proving viral circulation in humans (Human health problem)	“*And so we do not do tests, that's why we say there are no tests in humans, okay (…), but we don't look for them.”*
No exchange of information between partners	“*We are not used to exchanging information or plan an integrated surveillance: I would say there are multiple activities and these activities are not connected, and there are a few exchanges of information from all the different sides.”*
Early detection of the disease	“*I think the challenge is to be able to detect the reintroduction of the virus in our territories very quickly, and this detection is first of all through the animal surveillance system and mainly avian and equine.”*
Lack of testing	“*If the clinician does not ask for WNV testing, it is impossible for the laboratory to add it systematically in every syndrome, neurologic syndrome.”*

Some actors indicated that their concerns had not been fully addressed and felt excluded from the network, albeit having particular tasks inside the surveillance system (data collection, laboratory diagnostics) and sometimes do not understand some procedures related to their components due to a lack of information and communication with other actors.

Actors from the human health component have expressed that their main concern is the absence of human cases or limited information available on the impact of WNV on the human population of Guadeloupe. Physicians are fully alert and appear not to be aware of the disease, and they do not have any specific instructions from public health authorities to prescribe WNV tests. Consequently, they do not prescribe WNV diagnostic tests, so possible mild WNF cases could be mistaken for dengue fever due to similar symptomatology and a high prevalence in the region. Also, not all laboratories can carry out WNV testing, so samples would be sent to overseas reference laboratories in France, which hampers the physicians' prescriptions and the centralization of the results by the local laboratories leading to a loss of information.

## Discussion

### Constraints to Multidisciplinary and Cross-Sectoral Collaborations

These results show several limitations of the current WNV surveillance organization threatening the sustainability of the network. The SNA results show that flows of information between partners in Guadeloupe are constrained by the type of organization (or sector), a common practice where people communicate with those sharing the same working space or interests. WNV surveillance is mainly led by actors belonging to the animal health sector; they have created some links with specific human health actors, which in turn are connected to isolated members inside their sector. The environmental component is also underrepresented and ought to be an essential addition to the WNV surveillance system in the future. SNA results also show the unevenness of the communication with: two members being the central actors having most connections and representing the link between actors—they act both as hubs and authorities—while most actors are a part of the system because they are linked to only one person inside the network. In these cases, it would be hard to recover the communication routes established by these actors if either node disappears. This organization results from the absence of a formal protocol and a lack of governance that encompasses all sectors, where roles, responsibilities, and communication mechanisms between each organization or actor would be described. Actors need to communicate and collaborate directly with one another. Getting to know other actors by setting up meetings or trust-building activities is vital to create long-lasting connections that can be reflected in professional work and the shaping of efficient working groups. The “beer-and-pizza concept” mentioned by professor Craig Stephen is quite efficient in forming stronger groups: actors are encouraged to meet in relaxed environments, thus building friendly relationships (33). In the south of France, annual meetings of the WNV surveillance in the early 2000s used to be organized in the heart of the Natural Regional Park of Camargue. They included a field trip, bird watching, and other activities, allowing actors from all sectors to meet and get to know each other in a preserved, unique environment, that was also the virus's playground. With the current COVID-19 pandemic, this might be a bigger challenge. However, there may be something innovative to do, bearing in mind that collaboration needs to be mainly understood as a human process and not an obligation inside the professional area. Furthermore, an organizational structure is desirable where actors can get to know one another, their work line, and possible contributions to the network. Trust and established relationships can make the difference in communication.

Anticipating the considerable limitation of information sharing in a context where partners are willing to share information, the WNIS, and R Shiny platform are expected to fill major gaps by providing supporting infrastructure to facilitate the exchange of information and knowledge between partners. Moreover, it is expected to facilitate integrated data analysis, the definition of new research and surveillance questions, and the coordination of future WNV surveillance and research activities. If well-adopted, it may contribute to developing a systemic organization (polycentric, highly connected, and multidimensional), especially if it is extended to other vector-borne and zoonotic diseases.

With the advent of new information and communication technologies and their democratization over the last 10 years or so, IS have become more efficient and reliable: standardization of information, speed, reliability, availability, security, and shareability are all criteria for performance and optimization, which means that today IS are key tools in monitoring systems (39). However, it is essential to parallel an in-depth study of existing constraints (human resources in terms of skills, budgetary constraints, and technical constraints depending on the study site) so that the deployment of an IS based on new technologies thrives in the long-term.

Other than communication issues, concomitant participation in Gruel's study (47) on key attributes of “One Health” implementation highlights several other major weaknesses limiting the successful implementation of the “One Health” approach in the WNV surveillance network. There is a lack of formal “One Health” governance, coordination, and monitoring mechanisms. Moreover, there is no synergistic pooling of resources, no integrated data analysis, and a general lack of awareness of the “One Health” approach with an under-recognition of “One Health” professionals' roles. In addition, actors—mainly those from the public health sector—mentioned that WNV is not a priority and has fewer funding opportunities compared to other health threats like Leptospirosis, dengue, and currently Covid-19, which have a most significant public health impact in Guadeloupe.

Having a wider “One health” integrated surveillance network to establish collaborative programs to prevent and tackle diseases would probably be a good option, especially in a challenging environment like Guadeloupe where the turnover of persons in research/health agencies is frequent, the system is fragmented, and resources are scarce. Finally, Guadeloupe is distant from mainland France and communication between local and national epidemiological surveillance systems is inconsistent.

### Implementation of a Pilot Integrated Surveillance in Guadeloupe

A strategy has been envisioned with the actors and recommendations were formulated during the final workshop to keep the WNV surveillance and research activities going on after this study, and shape the network on the longer term, linked at the national level, as overseas territories are generally poorly integrated.

WNV integrated surveillance in many European countries involved creating strong, well-integrated teams (36, 38, 48), which is still lacking in Guadeloupe. For this reason, the main short-term goal is to enhance collaboration and communication around the WNIS, the R shiny platform, and the use of partners' information to improve early warning systems for WNV based on sentinel animals. Big expectations have been set on these new technologies and are yet to be used regularly. An epidemiologist has been recruited for 1 year to moderate and facilitate communication within the network. She will operationalize the WNIS and conduct the integrated data analysis with the partners to increase WNV knowledge in Guadeloupe, posing new surveillance and research questions.

Specifically, integrated data analysis will allow identifying correlations between environmental variables and WNV circulation data in horses and poultry as well as with mosquito vector dynamics in Guadeloupe. Several environmental variables, including climatic factors, such as warmer temperatures at the beginning of the mosquito breeding season, landscape structures (comprising water bodies or higher normalized difference vegetation index), and reservoir bird ecology were shown to shape WNV circulation risks in different areas (49). These have been integrated into WNV models that analyze vector and host abundance spatially and more precisely map areas at-risk for WNV infections in Europe, the Mediterranean basin, and North America (50–52). Identifying such factors that are critical modulators for WNV circulation in Guadeloupe is a prerequisite for WNV risk modeling and mapping and anticipating future virus epizootics or epidemics.

Also, training workshops for partners on the use of the WNIS, databases, data visualization, and the surveillance system will be continued. Alongside, actors have identified four priority actions they will work on throughout 2021: (a) the governance and information sharing mechanisms, (b) surveillance protocols and links with the French Epidemiological Surveillance platform (ESA, https://www.plateforme-esa.fr/), (c) improvement of WNV and flavivirus diagnosis in humans in collaboration with the veterinary research diagnostic laboratory and the national reference laboratories for human and animal vector-borne diseases, and (d) databases and integrated data analysis, with the development of a consortium agreement.

Despite their little availability and constraints, all actors are eager to participate in the WNV surveillance amelioration with minor additional costs and want to collaborate and learn about what other actors have discovered regarding WNV, and that has not been published yet. In the future, the combined use of technology for disease surveillance and IS might give us a better idea of current disease circulation, providing strategies for the implementation of prevention and disease control programs in Guadeloupe (39). This integrated surveillance system could also be expanded to other mosquito-borne diseases or emerging zoonosis of concern.

It is also essential to address open questions about the presentation of WNF in humans in Guadeloupe. Public health experts with a background in neurology and diagnostics mentioned that the current sampling strategy is not adequate, and the lack of positive cases acts as a barrier for the detection of the pathogen in the human population. This is also related to a lack of disease awareness or WNV not being a priority for human health organizations. The budget for WNV surveillance is very limited and only intended to fund diagnostic assays. It is crucial to increase awareness of the existence of WNF in the region among clinicians to promote testing for the disease and gain epidemiological knowledge on the true burden of WNV in humans. Also, adding WNV testing to the prescription of patients that present dengue-like symptoms could help in identifying human WNV positive cases.

Currently, the environmental sector is not represented in the WNV surveillance network. Ecologists from the University of Antilles (UA), as well as several naturalist associations (ornithologists, bat specialists), are working with CIRAD and several institutes on a research project called, “Insula” (2020–2023), funded by the European Union and the Guadeloupe region. This project aims at studying the impact of biodiversity degradation of several ecosystems on the risks of transmission of vector-borne diseases in plants, animals, and humans. It was suggested to organize a collaboration between the WNV surveillance network and the Insula research project to organize mutually beneficial collaborations: pool some resources, organize joint activities, share relevant data/samples, etc. It is anticipated that this will help WNV surveillance get highly relevant data and information from wildlife and the environment until the French Office for Biodiversity in Guadeloupe starts its operations on wildlife disease surveillance.

Linking research and surveillance on emerging zoonotic diseases has always been considered essential both by research institutes and organizations in charge of public and animal health surveillance, however, it has historically been poorly operationalized. With the implementation of the “One Health” approach in Guadeloupe since 2019, the partners of the MALIN Project are pushing for a strong “One health” project aiming at strengthening research and surveillance of emerging health problems, including zoonotic/vector-borne diseases within the framework of the next European Research Development Funds (ERDF) program (2022–2027).

It would be interesting to include cycles of internal evaluations with feedback of the actors to adapt network operations to surveillance objectives and its governance regularly. In addition, evaluating the “one health-ness” of the WNV integrated surveillance in the near future would be important to see the degree of evolution of the operationality of the “One Health” approach. For this, several methodologies can be used to evaluate interventions like SNA and focused ethnography, as well as tools of the Network for the Evaluation of One Health (NEOH) (53) or the method proposed by Gruel et al. (47).

In the longer term, thanks to the collaborative efforts and future programs under development, we hope to enhance linkages with other French, EU, and global initiatives to prepare for pandemics and zoonosis prevention. Currently, the WNV surveillance in Guadeloupe is disconnected from the national level. Indeed, although the French Ministry of Agriculture and the French Ministry of Health are aware of the results of the WNV surveillance conducted in Guadeloupe in their respective animal and human population, other groups involved in the national WNV surveillance do not know much about WNV surveillance conducted in Guadeloupe and, more generally in other French overseas territories. Options to better integrate WNV information from Guadeloupe at the national level were discussed like establishing a formal communication between the WNV surveillance locally and the ESA platform “WNV group.” Also, developing a surveillance protocol for the Wild bird component and defining the access to the “Epifaune” national database of the OFB monitoring wild bird mortality (https://ofb.gouv.fr/le-reseau-sagir) and mechanisms to inform the local WNIS need to be defined.

WNV is a pathogen that, even if silent in most Latin America and the Caribbean, is still circulating in the region, with strains continuously evolving in North America (11). It remains unclear if WNV strains are regularly introduced in Guadeloupe or if there is an episodic circulation of a local strain. In the case of WNV in Guadeloupe, many of the ecological cycle components have not been discovered yet, so the findings associated with the WNV surveillance in all components will be key to understand better the epidemiology of WNV in the Neotropics.

Also, a big concern within the “One Health” approach, in general, is a lack of experts in social, legal, and economic sciences (31). In the WNV surveillance system in Guadeloupe, not even one social scientist was mentioned or known to be a part of the system. Besides, the social sciences' expertise could be very useful in the future, providing insights into the population's needs and how the network can expand and communicate. When incorporating the human component, there is a strong connection between social and ecological factors that can make the difference in disease transmission and be taken into account while making prevention, control, or surveillance programs (54, 55). Involving actors belonging to non-profit or community organizations is vital because they are usually engaged in programs with the general population. Because of this interaction, they can act as key actors in creating programs that may impact health in the region (56). As well, links with policy-makers would be essential to make long-lasting changes in the health of Guadeloupe.

Finally, competencies outside the fields of science and health are an essential addition to “One Health” programs. Leadership and horizontal management are needed to manage a broad range of complex issues, to create and evaluate new partnerships and collaborations, and integrate the knowledge of various stakeholders. Solution finding techniques, flexibility, communication skills, team building, and trust development are capacities that anyone who works in a multidisciplinary environment needs to practice and develop (57). Often overlooked, those competencies will have to be considered in future projects—either through capacity building programs or recruitments—to sustain the collaborative efforts.

## Conclusion

This work is one of the first collaborative works paving the way for subsequent “One Health” research and surveillance in Guadeloupe. It has started making improvements in communication and collaboration between actors of the WNV surveillance system, making actors aware of the existence of people that currently work in similar fields. With further improvements and changes to the network structure and organization, it might become a model of surveillance for other emerging zoonotic pathogens in Guadeloupe aiming to be resilient, which means able to respond to a crisis or adapt while keeping a strong and efficient communication. WNV circulation may be difficult to evidence in Guadeloupe, but actors need to be prepared for future threats of any type. Knowing one another and being already a part of a multidisciplinary team might reduce Guadeloupe's health vulnerability and make a difference in the course of a health emergency or an outbreak. Surveillance actors now have a tool to save time and money while building stronger relationships and inter-institutional cooperation along the way.

## Data Availability Statement

The raw data supporting the conclusions of this article will be made available by the authors, without undue reservation.

## Ethics Statement

Ethical review and approval was not required for the study on human participants in accordance with the local legislation and institutional requirements. The patients/participants provided their written informed consent to participate in this study.

## Author Contributions

JP designed, directed, coordinated, and organized all phases of the project. MG did the Social Network Analysis, interviews, and data collection from partners. DC and AD developed the information system and the R Shiny app. NP, LA, AV-R, and CH-S provided surveillance information and input from their sectors during the participatory workshops. MG and JP drafted the article. SL participated actively in the latest workshops. All authors contributed to the article, discussed results, participated in the workshops and meetings, and approved the submitted version.

## Conflict of Interest

The authors declare that the research was conducted in the absence of any commercial or financial relationships that could be construed as a potential conflict of interest.

## Abbreviations

ANSES: French Agency for Food, Environmental and Occupational Health & Safety
ARS: Regional Health Agency
CHU: Center Hospitalier Universitaire
CIRAD: French Agricultural Research Center for International Development
CIRE: Interregional Epidemiology Unit of Antilles-Guyane
DAAF: Direction de l'Alimentation, l'Agriculture et la Forêt
IPG: Institute Pasteur de Guadeloupe
IS: Information System
OFB: French Agency for Biodiversity
SNA: Social Network Analysis
SPF: Santé Publique France
WNND: West Nile Neuroinvasive Disease
WNV: West Nile Virus
WNIS: West Nile Information System.
